# Supplementary material for: Multi-omic data integration enables discovery of hidden biological regularities
Source: Nat Commun. 2016 Oct 26;7:13091. doi: 10.1038/ncomms13091 (PMC5095171; doi:10.1038/ncomms13091)
Supplement: Supplementary Information — Supplementary Figures 1-9, Supplementary Notes 1-5, Supplementary Methods and Supplementary References [file ncomms13091-s1.pdf]

Multi-omic data integration enables discovery of hidden biological regularities - Supplement

Supplementary Figures

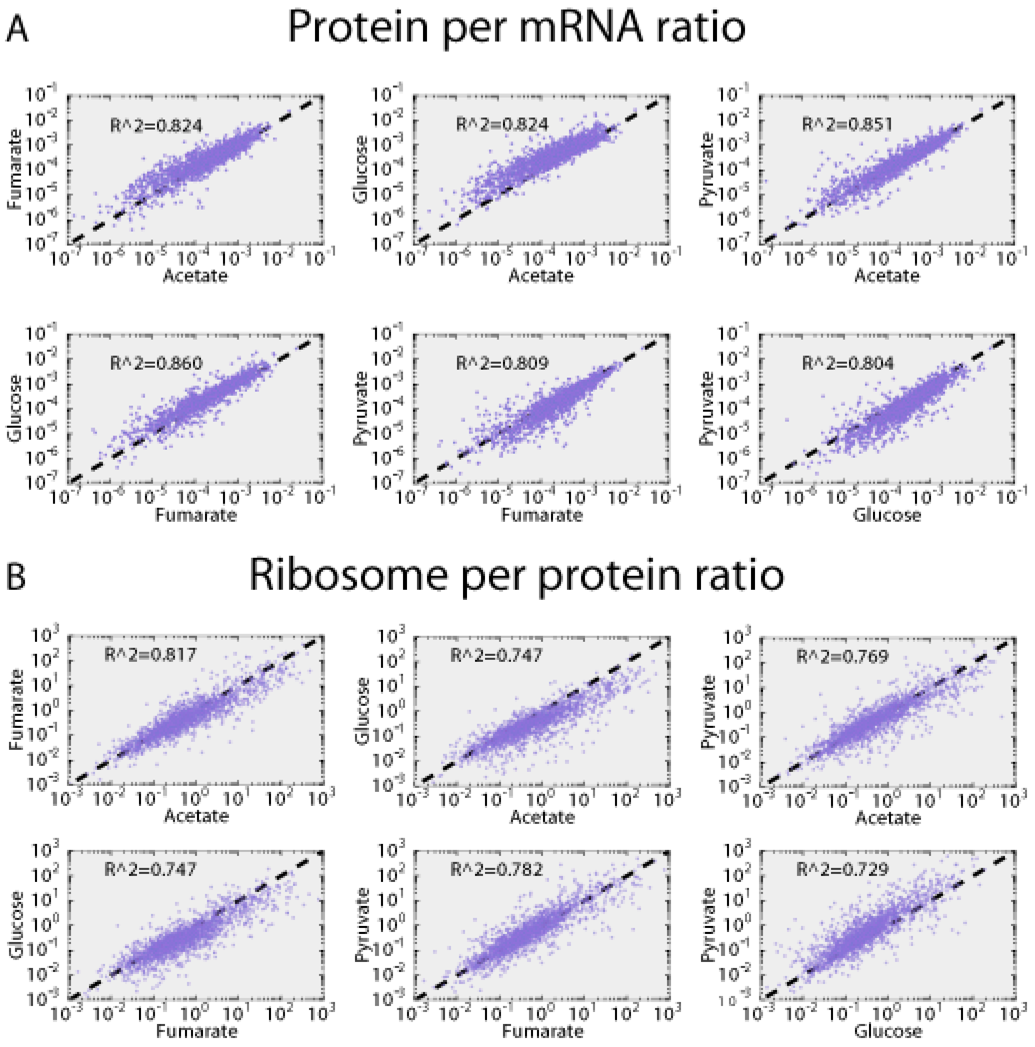

Supplementary Figure 1: Protein per mRNA ratios (A) and ribosome per protein (B) ratios across environments are highly conserved.

Ratios span several orders of magnitude across genes, but are highly conserved across different experimental conditions

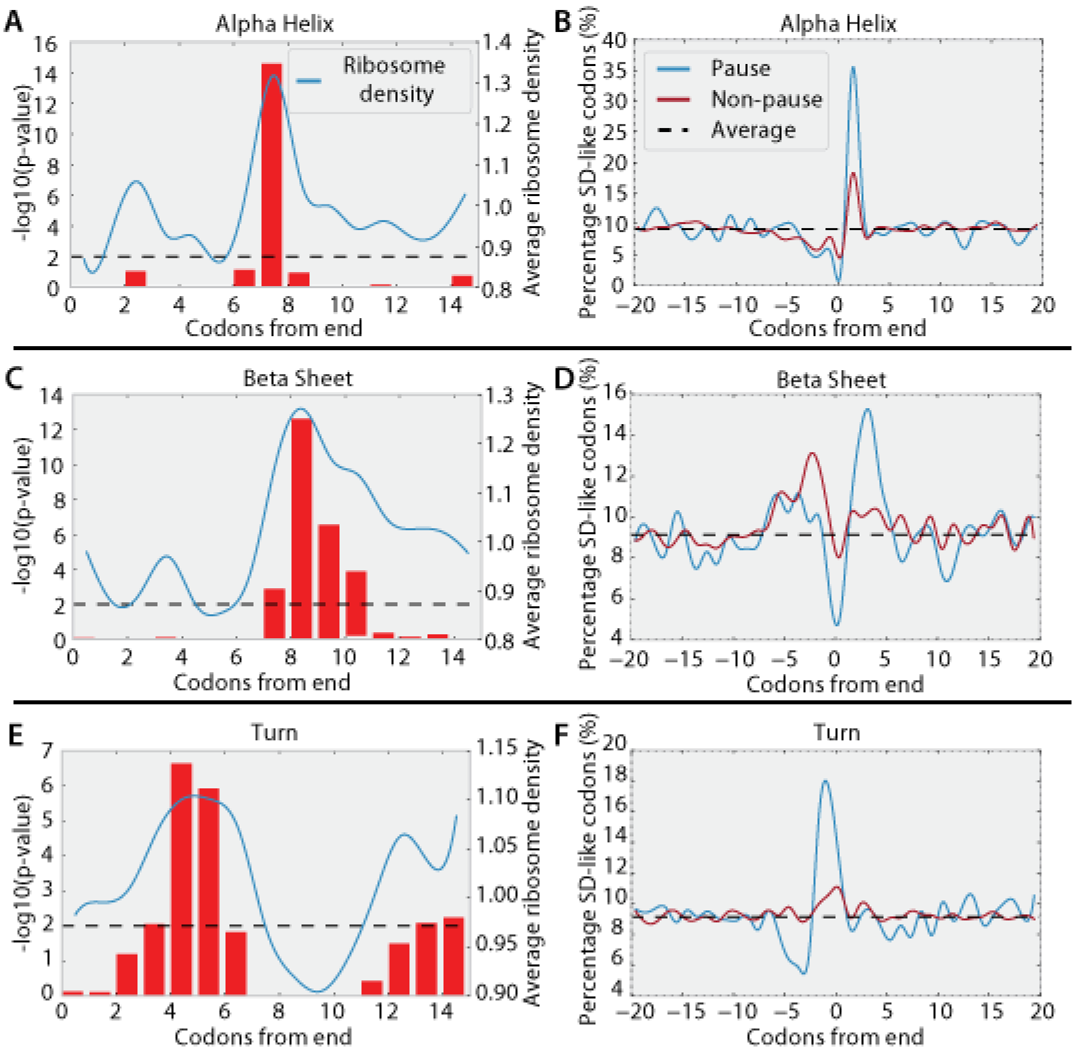

Supplementary Figure 2: Pause site enrichment and percent SD-like sequence near ends of secondary structures.

Hypergeometric enrichment testing was used to determine locations downstream of secondary structures which were enriched for pausing (A, C, E). Red bars represent p-value of enrichment. Blue line indicates the per gene normalized ribosome density at each codon position averaged across all occurrences of the secondary structure, showing a matching increase in average ribosome density. Based on the codon locations indicated by the enrichment test, all occurrences of each secondary structure were divided into those with corresponding pause sites (Pause) and those without (Non-Pause). The percentage occurrence of Shine-Dalgarno-like sequences appearing near the ends of these secondary structures is shown (B, D, E). The global average occurrence of an SD-like sequence is 9.13%, but this increases greatly at certain codon locations near the ends of secondary structures with corresponding pause sites (Blue line).

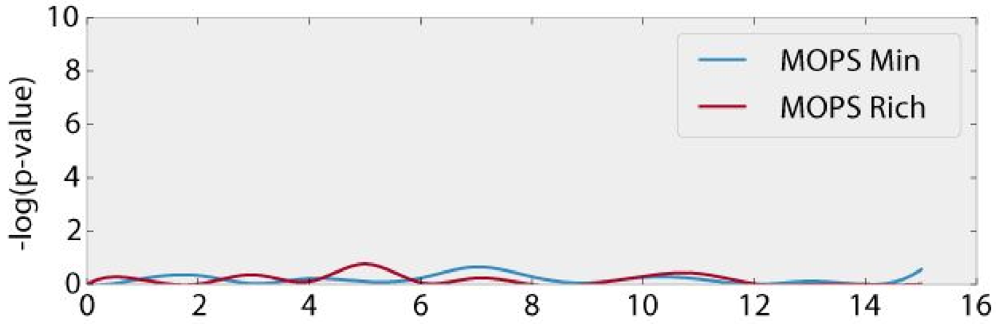

Supplementary Figure 3: Hypergeometric enrichment test of codons downstream from annotated SCOP domains.

No enrichment was found when codons downstream from the ends of domains were tested for pause site enrichment. Previous studies have found evidence that show slow translating regions between domains might be important for proper folding. However the location and even presence of pause sites might only occur on a case by case basis specific to particular domains, and fail to show enrichment when tested on the genome scale.

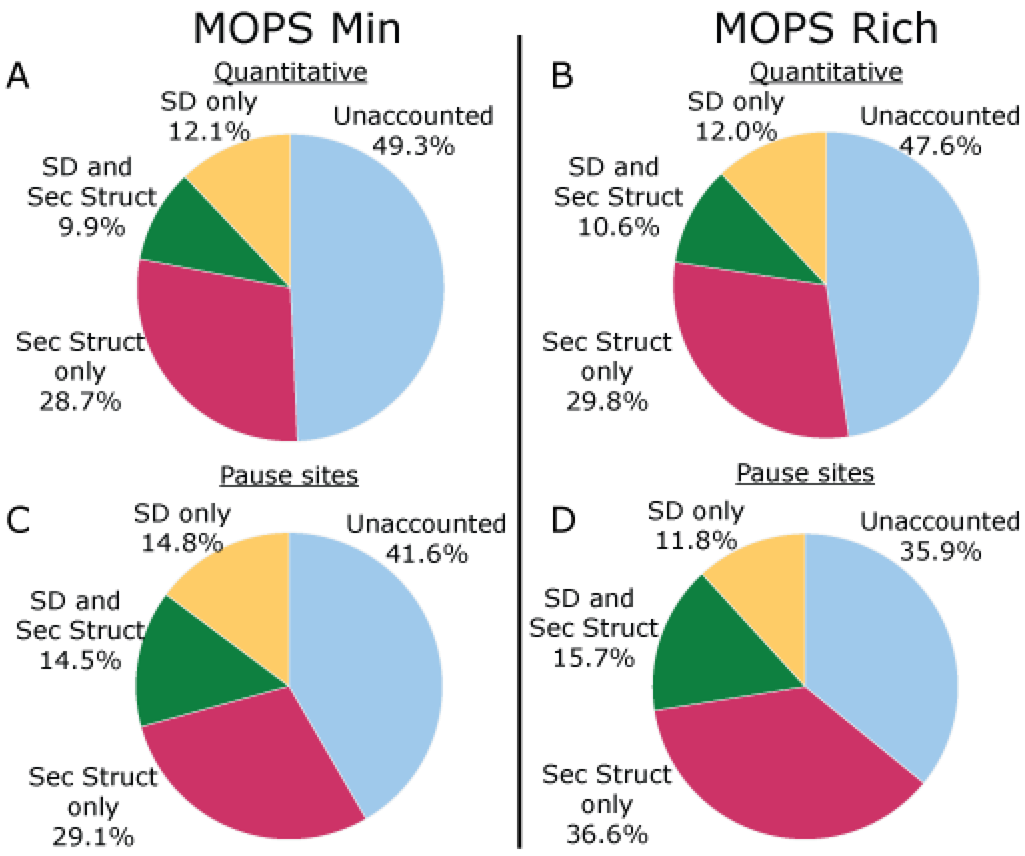

Supplementary Figure 4: Percentage of ribosome density and pause sites linked to SD-like sequences and/or Secondary Structure

Pie charts showing the distribution of pause sites linked to secondary structures and/or Shine-Dalgarno-like codons. In both MOPS minimal and MOPS Rich media, codons indicated to be pause-enriched for SD-like sequences accounted for around 20% of ribosomal density (A, B) and 30% of pause sites (C, D), while secondary structures accounted for 40% and 35% respectively. Of these, around 20-25% of these codons are indicated by both SD-like sequences and secondary structures. Around 50% of ribosomal density and 40% of all pause sites were still unaccounted for, indicating that there are other factors linked to pausing which were not included.

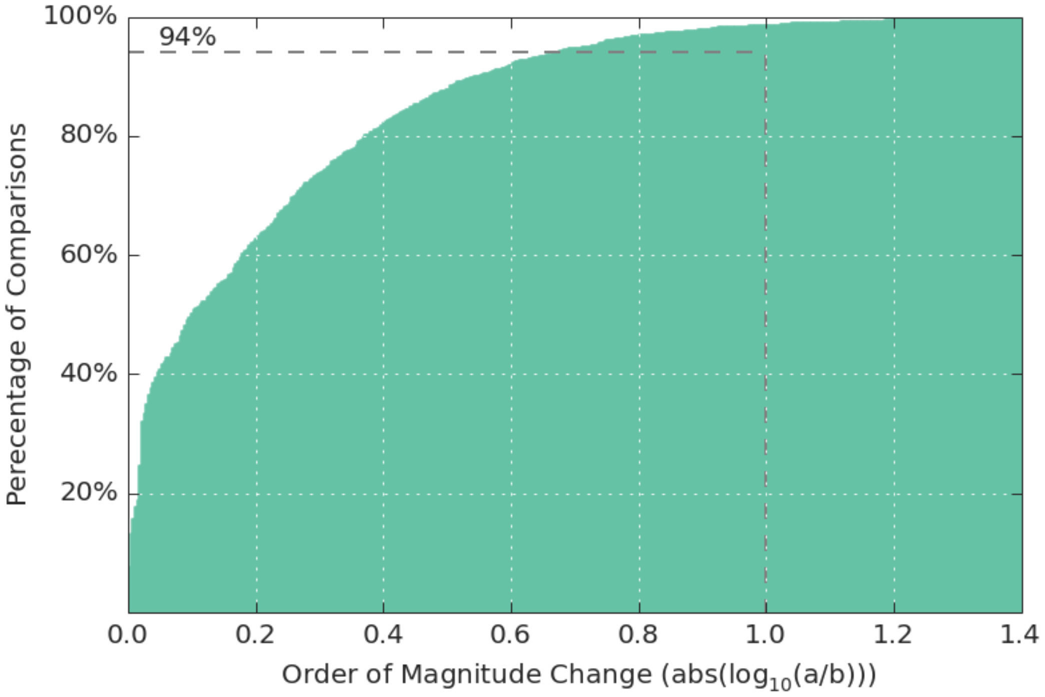

Supplementary Figure 5: Distribution of pairwise comparisons between computed k_{eff}

Each of the 284 k_{eff} was compared between conditions (6 comparisons between each of the 4 conditions). Plotted is the cumulative distribution of all these pairwise comparisons in terms of the change in order of magnitude. We observe that 94% of these comparisons remain within an order of magnitude. For the comparisons which were not within an order of magnitude, proteins associated with those complexes were more likely to catalyze multiple reactions (42.5% catalyzing more than one reaction v.s. 27.8% catalyzing more than one reaction).

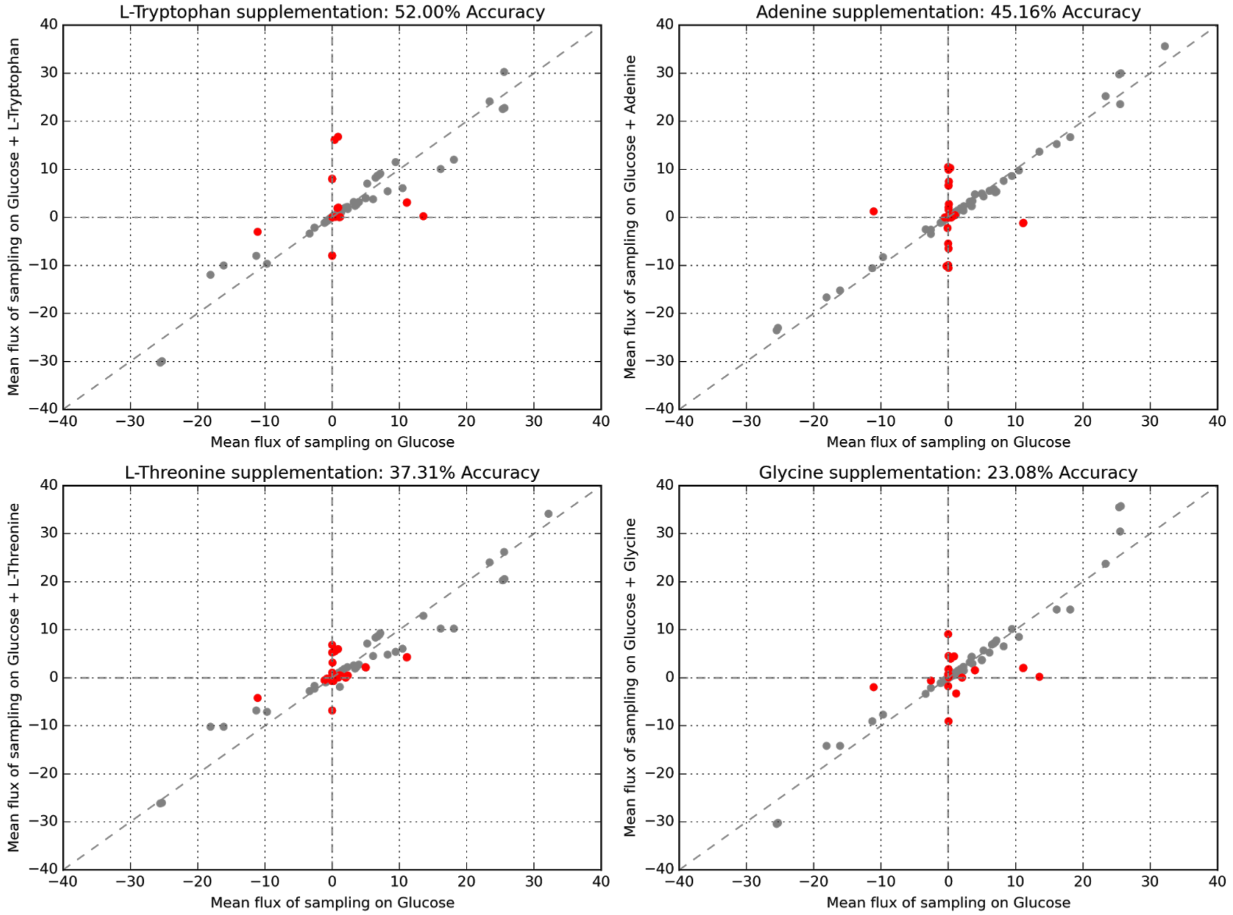

Supplementary Figure 6: Sampling *i*JO1366 Flux States Following Nutrient Supplementation

To compare ME predictions of gene differential expression to that of the M model, sampling was run on *i*JO1366 to identify reactions which changed significantly in flux (colored red) after supplementation. The model gene reaction rules were used to convert these to gene expression predictions, which were compared to mRNA sequencing to give the reported accuracies.

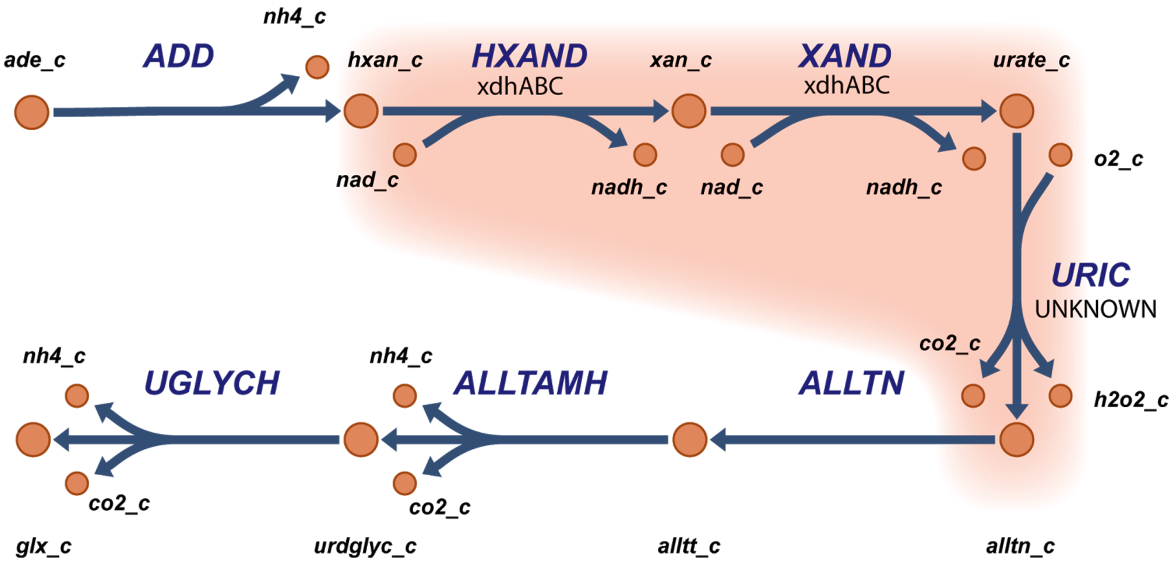

Supplementary Figure 7: Updates to the Adenine Degradation Pathway in the E. coli Metabolic Reconstruction.

As reconstructed in iJO1366 and iOL1650-ME, this pathway breaks down intracellular adenine to glyoxylate. However, our expression-profiling data with adenine supplementation suggests that the highlighted reactions do not occur to break down adenine, even though the upstream reaction adenine deaminase does occur. While these reactions were included in the model based on their homology to adenine degradation pathways in other organisms, it is likely that the pathway is latent or inactive in E. coli K-12. Therefore, the reactions HXAND and XAND were disabled during simulations on Adenine. Additionally, use of the unexpressed gene focB was also penalized.

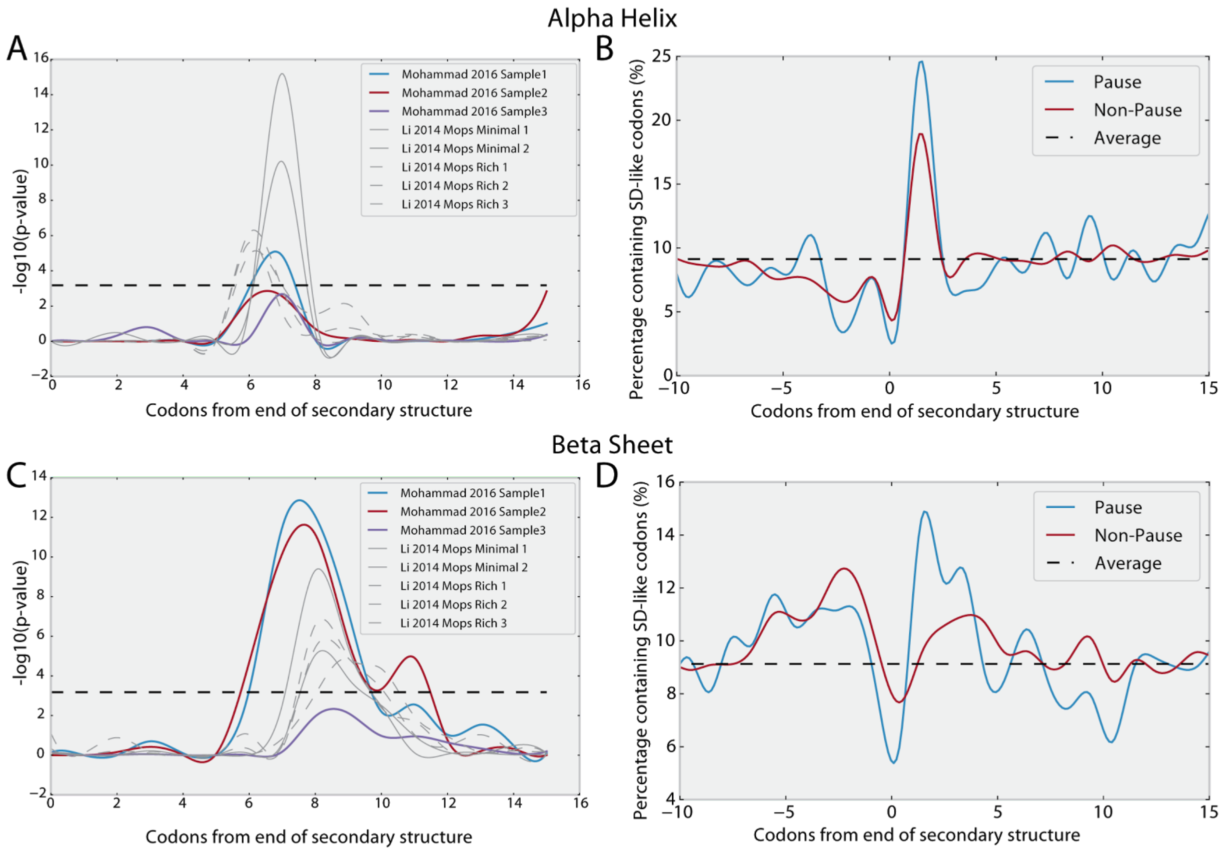

Supplementary Figure 8: Pause site enrichment downstream of secondary structures across datasets.

The location of pause site enrichments downstream of alpha helix (A) and beta sheet (C) secondary structure motifs between several datasets from Mohammad *et. al.* 2016 [1] and Li *et. al.* 2014 [2], which make use of different protocols. The location of pause site enrichment is consistent across both protocols. Similar to the analysis done in Supplementary figure 2B, D, F, the datasets from Mohammad *et. al.* 2015 also show increased prevalence of SD-like codons at the ends of secondary structures with pause sites downstream as compared to those without (B, D).

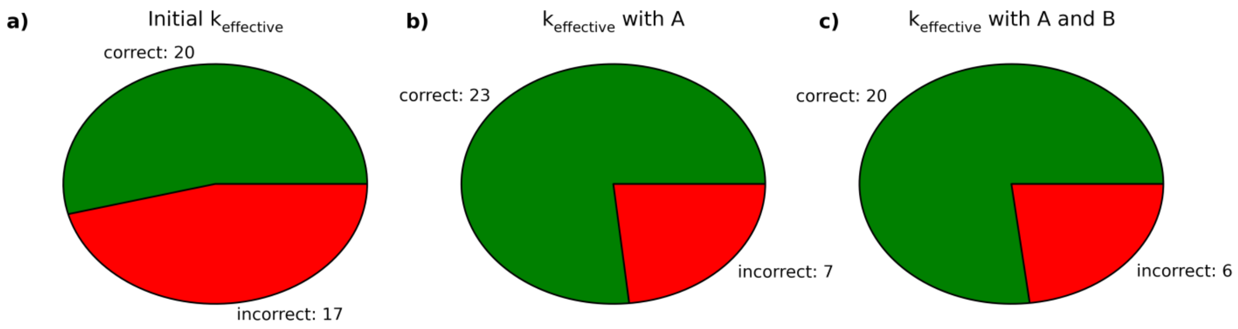

Supplementary Figure 9: Predicted Differential Expression between Fumarate and Acetate

The ME model was used to predict differential expression between growth on fumarate and growth on acetate as the main carbon substrates. These predictions were run with three different sets of k_{eff} and then validated using mRNA sequencing. This is described in more detail in the "Predictions of mRNA expression under identical conditions to proteomic data" section.

Supplementary Notes

Supplementary Note 1: Ribosome profiling pause site analysis

It has been established that translation rate is not constant along a gene. This has been demonstrated in-vitro using proteins such as firefly luciferase[10,11] and epoxide hydrolases[12]. Various phenomena such as codon usage[10,13], mRNA secondary structure[14], anti-Shine-Dalgarno-like sequences[15], poly-proline stretches[16] as well as protein domains[12,17,18] have been implicated in determining the locations of these pause sites. Slower translation rate have been shown anecdotally to improve solubility of heterologously expressed proteins[10,12] as well as improve yield and function[11,19], and has been implied to be necessary for proper co-translational folding[20–23], yet at the same time impose a fitness cost on the cell[24]. Here we show a link between secondary structure motifs, anti-Shine-Dalgarno-like sequences, and pausing locations along the length of a transcript.

Hypergeometric enrichment testing of the pause sites downstream from the end of each structure points towards the enrichment of pausing at certain codon locations (Supplementary Figure 2A, C, E). At the same time, sequence analysis near the ends of the secondary structures which have pause sites at these locations show increased occurrences of anti-Shine-Dalgarno-like sequence, approximately 6 codons upstream from the enriched pause site for each secondary structure motif (Supplementary Figure 2B, D, F). This indicates that secondary structures tend to have pause sites downstream from their ends, and anti-SD-like sequences might be used to induce the pausing. It is noted here that in contrast to other studies which determine the pausing propensity with relation to the assumed A site, assigning ribosome density to the 3' end of the read results in a pause site which is around 2-3 codons further downstream. For example, Li *et. al.*[15] found that anti-SD-like sequences were linked to pausing 8-11 nucleotides (~3rd-4th codon) downstream, which in our analysis occurs on the 6th codon downstream instead because of the different positional assignment of the read. Datasets from Mohammad *et. al.* 2016 which make use of an altered protocol in order to reduce biases also showed pause site enrichments at similar location, and show the corresponding increase in SD-like sequences near the ends of secondary structures (Supplementary Figure 8).

Overall, across both MOPS minimal and MOPS rich growth conditions, around 50% of ribosomal density and 60% of pause sites can be attributed to either anti-SD-like sequences or secondary structure motifs (Supplementary Figure 4). While this indicates that these two factors play a major role in ribosome pausing locations, the overlap between them is only between around 15% of all pause sites. This suggests that there are other factors unaccounted for in this study, which either require, or induce pausing, and should be the subject of further investigation.

Supplementary Note 2: Structure of ME models

Here we briefly introduce key formulations of the ME model, and refer the interested reader to a more complete supplementary information in O'Brien et. al. 2013[3]. The ME model is a steady state growth model which accounts for metabolism (M) and gene expression (E). Based on an input of available

nutrients to the cell such as carbon and nitrogen sources, it predicts: a) the cell's maximum growth rate in a specific condition, and at the maximum growth rate, b) metabolite uptake and excretion rates, c) metabolic reaction fluxes, and d) gene expression fluxes such as translation and transcription rates. This is achieved through formulating transcription, translation, transport and metabolic fluxes into a quasi-linear problem and solving for maximum growth rate, taking into account compartmentalization of proteins and metabolites into the cytoplasm, periplasm, and extracellular region. This is done as follows:

1. The metabolic network is described by a stoichiometric matrix, similar to those used in M-models[4] where rows represent metabolites and columns represent reactions. Coefficients represent the metabolites consumed (negative value) or produced (positive value) in each reaction. At steady state, there is no change in metabolite concentrations, hence we get:

$$S \cdot v = 0$$

where *S* is the stoichiometric matrix, and *v* is the flux vector, allowing us to solve for *v*.

2. In the ME model, translation is accounted for by enforcing that proteins have to be produced for all enzyme-catalyzed reactions, proportional to the flux for that particular reaction. Protein synthesis is balanced by protein dilution (due to cell division), which is proportional to the amount of protein required to hold the predicted flux. Note that in this case we disregard direct protein degradation as it has been shown to negligible in a growing *E. coli* cell for most metabolic genes[5,6]. While active degradation is important for many signaling proteins such as transcription factors, these proteins are a very small proportion of the whole proteome in rapidly growing cell. The depletion of most of the modeled proteins is therefore mostly through dilution caused by growth, and the depletion rate is simply the growth rate. Mathematically, this is represented below:

$$v_{translation,\,i} = v_{dilution,\,i} = \mu[E_i]$$

$$v_{reaction,\,i} = k_{eff,\,i}[E_i]$$

$$v_{translation,\,i} = \frac{\mu}{k_{eff,\,i}} v_{reaction,\,i}$$

The above relationships link the required translation rate $v_{translation,\,i}$ to the flux through the reaction $v_{reaction,\,i}$ as well as the predicted growth rate μ and the proteins effective catalytic rate $k_{eff,\,i}$. Unlike traditional M models where biomass has to be explicitly modeled, this inherently takes protein and macromolecule dilution into account during growth, and allows prediction of optimal protein production, and hence gene expression.

It is important to note that, in previous ME models[3], the effective catalytic rate was set to be proportional to the respective enzyme's solvent accessible surface area (SASA). Here, we make use of condition-specific proteomics data, which vastly improves this parameter and the predictive scope of the model itself. More details of this parameterization procedure is found in the following section.

Finally, translation of proteins are catalyzed by ribosomes, requiring tRNA and its synthethases and the cost of production of these molecules are also explicitly accounted for in the ME model. Analogous to metabolic enzymes, ribosome efficiency k_{ribo} is a necessary parameter for coupling the ribosome production rate to the overall proteome translation rates, and is shown below:

$$v_{synthesis,\ ribosome} = v_{dilution,\ ribosome} = \sum_i length(peptide_i) * \frac{\mu}{k_{ribo}} * v_{translation,\ i}$$

Because ribosomes are themselves partially made up of peptides which need to be synthesized, this coupling creates an asymptotic relationship between ribosome production and growth rate. Placing a limitation on cell size replicates the natural phenomenon where growth rate is constrained by the balance between enzyme and ribosome production even in the overabundance of nutrients.

3. In the ME model presented in this contribution, transcription is now achieved through the production of mRNA from nucleotides, catalyzed by RNA polymerase. This is also handled in a similar way to ribosomes and metabolic enzymes through dilution of RNA polymerase, proportional to the total flux through all transcription reactions.

Supplementary Note 3: ME model coupling parameters

In addition to metabolic reactions, the ME model describes various biological processes as biochemical reactions. For example, translation of a protein is described by a reaction assembling the component amino acids into peptides. An enzyme complex is then formed by a reactions which assemble together the various peptides and cofactors, and more reactions which apply the necessary post-translational modifications. Dependent reactions are linked through what are termed “coupling constraints” because they force a certain amount of flux through one of the reactions based on the level of flux through the other, effectively coupling the processes. For example, flux through a metabolic flux is coupled to production of the catalytic enzyme. A translation reaction is coupled to both reactions which produce ribosomes and reactions which transcribe the mRNA. In iOL1650-ME, these coupling constraints are expressed as inequalities as functions of growth rate μ to reflect how the nature of many of these constraints are nonlinear in growth. However, the constants in these functions are set for each individual coupling constraint. A detailed description of all the processes and coupling parameters in the iOL1650-ME model is available in the supplementary information of that manuscript[3].

One of the most critical coupling constraints to the function of the ME model is the k_{eff}, which describes the amount of enzyme required on average to sustain a unit of flux under in vivo conditions. An example coupling constraint for a metabolic reaction has the form:

$$v_{metabolic} \leq \frac{k_{eff}}{\mu} v_{enzyme}$$

This coupling constraint expresses both how as growth rate increases, more enzyme must be made to pass on to each daughter cell, and how the amount of enzyme production relates to its efficiency through the k_{eff} parameter. While the k_{eff} parameter has units of 1/time like a k_{cat}, it is strictly less than the k_{cat}, as it is describing the amount of enzyme which must be made to catalyze a unit of flux instead of the maximal flux a particular enzyme can sustain. The reason this parameter is critical to the function of the ME model is because it describes the relative cost of each enzyme. An appropriate analogy is an econometric model. Where the reactions themselves express operational costs, and the cost of the enzyme is the capital cost. A good example of this the difference between the “expensive” but efficient pyruvate dehydrogenase complex compared to pyruvate formate lyase. Pyruvate dehydrogenase (PDH) and pyruvate formate lyase (PFL) both convert pyruvate to acetyl coA to allow carbon to flow through the TCA cycle. Flux through PFL will result in secretion of formate, whereas flux through PDH will result in

production of CO_2, which is the *in vivo* behavior. In *E. coli*, PDH is one of the most expensive metabolic enzymes because it consists of 60 subunits. However, an optimally efficient *E. coli* cell might still produce this enzyme over PFL because its catalytic rate is more than commensurately higher, as it exploits phenomena such as substrate tunneling and multiple catalytic sites, and will therefore have a lower protein cost per unit of flux catalyzed. Therefore, in order for an ME model to correctly predict flux through PDH, it must have k_{eff} parameters for PDH and PFL which represents this tradeoff accurately. In a genome-scale ME model, these parameters affect the cost between all the various alternate pathways and isozymes. Therefore, the accuracy of the model predictions of protein expression and the physiological state of the cell will depend on reasonable relative values of these parameters.

Supplementary Note 4: Simulation of Batch Growth with ME

One of the advantages of the ME model over traditional M models is its ability to account for proteome limitation. This gives the model the ability to correctly simulate batch growth, where a cell has a surplus of nutrients around it, but is limited by its ability to produce the proteins which are required to process these nutrients. Unlike M models, which can predict growth rates given a specific substrate uptake rate, a ME model can predict the maximal growth rate given an unbounded substrate uptake (allowing the model to take up as much substrate as it wants). O'Brien et al. investigated how proteome limitation begins to take effect as the substrate uptake rate approaches the optimal value, eventually causing a maximal growth rate[3]. Beyond this optimal growth rate, the model is infeasible with any substrate uptake rate because of the proteome limitation constraints. This optimal growth rate can be computationally identified by doing a binary search. Because the experimental data used in this study were all generated under batch growth conditions, the simulations of growth used this batch growth procedure, which computes a proteome-limited state.

Supplementary Note 5: Simulating ME with estimated parameters

In the absence of in vivo experimental measurements, the original iOL1650-ME model estimated k_{eff} values based on the solvent-accessible surface area (SASA), which is a function of protein size[3,7]. We sought to evaluate the effect of our new set of estimated parameters values on predictions of differential gene expression. We simulated a switch of the primary carbon substrate from fumarate to acetate, which could be validated by the previously generated mRNA sequencing data sets (GSE59759). Using the original k_{eff} values obtained from protein size based estimation we observed a significant number of false positive predictions due to incorrect pathway usage, with 17 false positives found out of 37 total predictions. Using the consensus k_{eff} parameters derived from sets A + B yielded only 6 false positives with the same number of correct predictions. Interestingly, almost all of the improvement in prediction came from using parameter set A alone (Supplementary Figure 9), which constrained parameters in central carbon metabolism. This result suggests that the accuracy of a ME model is most sensitive to these key 28 k_{eff} parameters which lie in its high flux backbone[8].

After showing that the set of estimated k_{eff} values gives better predictions for previously examined nutrient shift, we generated new experimental data for growth on dual substrates. We computed predicted differential gene expression after media supplementation with four key nutrients: Adenine, Glycine, L-Threonine, or L-Tryptophan, using 1) k_{eff} found in set A, and 2) k_{eff} found in both sets A and B

(Table 1). Genes that were computed to change in expression by more than a factor of 16 after supplementation were considered to be predictions of differential expression (Figure 4). Prediction validation was performed using mRNA sequencing data, some of which was taken from a previous study[9], to experimentally determine differentially expressed genes. Predictions were made with a slightly modified iOL1650-ME (Supplementary Figure 7). For all four supplementations, the accuracy of ME predictions (Table 1) was higher than those resulting from sampling M model flux states (Supplementary Figure 6). Moreover, the accuracy increased when comparing parameter set A + B to parameter set A alone. Using parameter set A + B, the accuracy of predictions of ranged from 55 to 100%, and all predicted sets were significantly enriched for differentially expressed genes ($p < 0.05$ using a hypergeometric distribution).

These results suggest that a parameterized genome-scale ME model, due to its incorporation of enzyme biosynthetic costs, gives a significant improvement in prediction of gene expression over existing methods using M models alone. For example, the ME model correctly predicts the often non-intuitive shift of amino acid precursors. Specifically, when supplementing with L-Threonine, the ME model will produce L-Serine from L-Threonine directly, as observed experimentally. On the other hand, the ME model predicts no significant up regulation of glyA to produce L-Serine from Glycine supplementation, as seen in the expression profiling data. The *i*JO1366 M-model will incorrectly predict this change (Figure 5c), demonstrating how the ability to account for protein expression costs improves the accuracy of the predicted flux state. Moreover, as a result of its quantitative numerical predictions of gene expression, ME models can also predict partial up and down regulation of genes, in addition to binary responses when a gene goes from active to inactive. For example, after supplementation with Adenine, the model correctly predicts downregulation of purHD and purMN (Figure 5c).

Supplementary Methods

mRNA seq

Cells were harvested at mid-log (OD600 ~ 0.3) in biological duplicates for each condition. From each sample, 3mL of culture were mixed with 6mL RNAprotect Bacteria Reagent (Qiagen), incubated for 5 minutes, and then centrifuged at 5000g for 10 minutes at room temperature. Total RNA samples were then isolated from the pellet using the RNeasy Plus Mini kit (Qiagen). Samples were quantified using a NanoDrop 1000 (Thermo Scientific) and an Agilent RNA 6000 Nano Kit with an Agilent 2100 Bioanalyzer. Strand-specific mRNA libraries were created using the dUTP method, with ribosomal rRNA subtraction with the Ribo-Zero rRNA Removal Kit (Epicentre). Libraries were run on a Miseq (Illumina, CA) multiplexed with 2 duplicates per run as per the manufacturer's instructions. Expression values were computed using the bowtie[25] and cufflinks[26] packages. The processed data were uploaded to GEO under accession numbers GSE59759 and GSE59760.

Structural Data Retrieval and Manipulation

Incorporating protein-related information into a GEM involves four stages of semi-automated curation: (i) map the genes of the organism to available experimental protein structures, found in publicly available databases, such as the Protein Data Bank (PDB); (ii) determine genes with and without available protein structures and perform homology modeling using the I-TASSER suite of programs[27] to fill in gaps where crystallographic or NMR structures are not available; (iii) perform ranking and filtering of PDB structures for each gene based on a set selection criteria (e.g., resolution, number of mutations, completeness); (iv) map GEM genes to other databases (e.g., BRENDA[28,29], SwissProt[30], Pfam[31], SCOP[32]) for complementary protein-structure derived data. The quality of the reconstruction expansion process to include high confidence protein structures is considered by carrying out a series of QC/QA verification steps during the ranking and filtering stage. The GEM annotation of the organism of interest is stored in SBML and Matlab formats and many organisms can be found in the BiGG database [33]. Amino acid sequence of the proteins of interest are stored in FASTA format. To map protein structural data to a GEM, we make use of Python modules, ProDy[34,35] and Biopython[36] to parse information in the PDB files. The molecular visualization software VMD[37] was used for viewing the 3D structure of the modeled protein and the predicted functional sites and the creation of images. Installation of PfamScan and HMMER3 algorithms are required for generating protein fold families for certain proteins[38,39]. Open source software for protein structural predictions are available and are used in conjunction with the IPython framework.

Predictions of mRNA expression in parametrized conditions

We sought to evaluate the effect of the estimated parameter values on predictions of differential gene expression under identical experimental conditions as the proteomics used to parameterize the model. We simulated a switch of the primary carbon substrate from fumarate to acetate, and obtained mRNA sequencing data (GSE59759) for E. coli K-12 BW25113 (obtained from the Coli Genetic Stock Center)

cultivated under identical experimental conditions to those of the proteomic data. Bach growth simulations were then performed using 3 different sets of k_{eff} parameters: the initial solvent-accessible-surface area parameters, k_{eff} parameters derived from fluxomics (set A), and k_{eff} parameters derived from fluxomics and our proteomics-based algorithm (set A + B). Predicted differential expression was compared to the set of genes identified as differentially expressed by cufflinks[26] at a false discovery rate of 0.05. In order to account for accuracy on a level field with respect to sensitivity, we varied the computational cutoff so that we would have a similar number of correct predictions (as close to 20 as possible), allowing us to directly compare the number of incorrect predictions. Using the the original iOL1650-ME model k_{eff} values obtained from protein size based estimation, we observed a significant number of false positive predictions due to incorrect pathway usage, with 17 false positives found out of 37 total predictions. Using a consensus k_{eff} parameter set derived from both experimentally measured flux values[40] (set A) and model-predicted values (set B) yielded only 6 false positives with the same number of correct predictions. Additionally, we were able to improve predictions greatly using the 28 k_{eff} parameter values from set A alone (Supplementary Figure 6), which only constrained parameters in central carbon metabolism. This result suggests that the accuracy of a ME model is most sensitive to k_{eff} parameters for reactions which lie along its high flux backbone.

Sampling of M-model flux states in iJO1366

The optGpSampler[41] software was used to sample flux states in the iJO1366 M model using its python API. First, the model was reduced by removing blocked reactions as identified by flux variability analysis in cobrapy[42]. For each simulation, the lower bound on the biomass reaction was set to 90% of its optimal value. Additionally, the reactions HXAND, XAND, and URIC were blocked (Supplementary Figure 8). The sampling algorithm was then run for 100 steps and generated 10000 points for each simulation. Afterwards, the fluxes were linearly scaled such that the mean flux of the biomass reaction was 1. Sampling was run on the model with only D-Glucose uptake, and also with 10 mmol/gDw/hr uptake allowed of each of the supplements. Reactions which were unbounded (as determined by an FVA maximum greater than 500 or an FVA minimum of less than -500) were excluded from the subsequent analysis. For each of the supplements, predicted changed reaction fluxes between the supplemented and unsupplemented samples were determined by finding reaction fluxes where (1) the mean changed by more than a factor of 2 and (2) the mean changed by more than the sum of the standard deviations for the supplemented and unsupplemented fluxes. These reactions were converted to gene predictions by assuming all genes in the gene reaction rule for the changing reaction were up or down regulated. These gene predictions were validated against mRNA sequencing data in the same manner as the ME gene differential expression predictions. This method was used instead of the more traditional method comparing pairs of samples as done in some other studies[9] because it resulted in higher accuracy than those methods with this data.

Supplementary References

1. Mohammad, F., Woolstenhulme, C. J., Green, R. & Buskirk, A. R. Clarifying the Translational Pausing Landscape in Bacteria by Ribosome Profiling. *Cell Rep.* **14,** 686–694 (2016).
2. Li, G.-W., Burkhardt, D., Gross, C. & Weissman, J. S. Quantifying absolute protein synthesis rates reveals principles underlying allocation of cellular resources. *Cell* **157,** 624–635 (2014).
3. O'Brien, E. J., Lerman, J. A., Chang, R. L., Hyduke, D. R. & Palsson, B. Ø. Genome-scale models of metabolism and gene expression extend and refine growth phenotype prediction. *Mol. Syst. Biol.* **9,** 693 (2013).
4. Orth, J. D. *et al.* A comprehensive genome-scale reconstruction of Escherichia coli metabolism--2011. *Mol. Syst. Biol.* **7,** 535 (2011).
5. Maurizi, M. R. Proteases and protein degradation in Escherichia coli. *Experientia* **48,** 178–201 (1992).
6. Nath, K. & Koch, A. L. Protein degradation in Escherichia coli. II. Strain differences in the degradation of protein and nucleic acid resulting from starvation. *J. Biol. Chem.* **246,** 6956–6967 (1971).
7. Miller, S., Lesk, A. M., Janin, J. & Chothia, C. The accessible surface area and stability of oligomeric proteins. *Nature* **328,** 834–836 (1987).
8. Almaas, E., Kovács, B., Vicsek, T., Oltvai, Z. N. & Barabási, A.-L. Global organization of metabolic fluxes in the bacterium Escherichia coli. *Nature* **427,** 839–843 (2004).
9. Bordbar, A. *et al.* Minimal metabolic pathway structure is consistent with associated biomolecular interactions. *Mol. Syst. Biol.* **10,** (2014).
10. Yu, C.-H. *et al.* Codon Usage Influences the Local Rate of Translation Elongation to Regulate Co-translational Protein Folding. *Mol. Cell* **59,** 744–754 (2015).
11. Siller, E., DeZwaan, D. C., Anderson, J. F., Freeman, B. C. & Barral, J. M. Slowing bacterial translation speed enhances eukaryotic protein folding efficiency. *J. Mol. Biol.* **396,** 1310–1318 (2010).
12. Hess, A.-K., Saffert, P., Liebeton, K. & Ignatova, Z. Optimization of translation profiles enhances protein expression and solubility. *PLoS One* **10,** e0127039 (2015).
13. Gardin, J. *et al.* Measurement of average decoding rates of the 61 sense codons in vivo. *Elife* **3,** (2014).
14. Sørensen, M. A., Kurland, C. G. & Pedersen, S. Codon usage determines translation rate in Escherichia coli. *J. Mol. Biol.* **207,** 365–377 (1989).
15. Li, G.-W., Oh, E. & Weissman, J. S. The anti-Shine-Dalgarno sequence drives translational pausing and codon

choice in bacteria. *Nature* **484,** 538–541 (2012).

16. Woolstenhulme, C. J., Guydosh, N. R., Green, R. & Buskirk, A. R. High-precision analysis of translational pausing by ribosome profiling in bacteria lacking EFP. *Cell Rep.* **11,** 13–21 (2015).
17. Zhang, G. & Ignatova, Z. Generic algorithm to predict the speed of translational elongation: implications for protein biogenesis. *PLoS One* **4,** e5036 (2009).
18. Zhang, G., Hubalewska, M. & Ignatova, Z. Transient ribosomal attenuation coordinates protein synthesis and co-translational folding. *Nat. Struct. Mol. Biol.* **16,** 274–280 (2009).
19. Zhou, M. *et al.* Non-optimal codon usage affects expression, structure and function of clock protein FRQ. *Nature* **495,** 111–115 (2013).
20. Saunders, R. & Deane, C. M. Synonymous codon usage influences the local protein structure observed. *Nucleic Acids Res.* **38,** 6719–6728 (2010).
21. Purvis, I. J. *et al.* The efficiency of folding of some proteins is increased by controlled rates of translation in vivo. A hypothesis. *J. Mol. Biol.* **193,** 413–417 (1987).
22. Komar, A. A., Lesnik, T. & Reiss, C. Synonymous codon substitutions affect ribosome traffic and protein folding during in vitro translation. *FEBS Lett.* **462,** 387–391 (1999).
23. Subramaniam, A. R., Zid, B. M. & O'Shea, E. K. An integrated approach reveals regulatory controls on bacterial translation elongation. *Cell* **159,** 1200–1211 (2014).
24. Hingorani, K. S. & Gierasch, L. M. Comparing protein folding in vitro and in vivo: foldability meets the fitness challenge. *Curr. Opin. Struct. Biol.* **24,** 81–90 (2014).
25. Langmead, B. & Salzberg, S. L. Fast gapped-read alignment with Bowtie 2. *Nat. Methods* **9,** 357–359 (2012).
26. Trapnell, C. *et al.* Transcript assembly and quantification by RNA-Seq reveals unannotated transcripts and isoform switching during cell differentiation. *Nat. Biotechnol.* **28,** 511–515 (2010).
27. Zhang, Y. I-TASSER: Fully automated protein structure prediction in CASP8. *Proteins: Struct. Funct. Bioinf.* **77,** 100–113 (2009).
28. Chang, A., Scheer, M., Grote, A., Schomburg, I. & Schomburg, D. BRENDA, AMENDA and FRENDA the enzyme information system: new content and tools in 2009. *Nucleic Acids Res.* **37,** D588–D592 (2009).
29. Schomburg, I. *et al.* BRENDA, the enzyme database: updates and major new developments. *Nucleic Acids Res.* **32,** D431–D433 (2004).
30. Boeckmann, B. *et al.* The SWISS-PROT protein knowledgebase and its supplement TrEMBL in 2003. *Nucleic*

Acids Res. **31,** 365–370 (2003).

31. Bateman, A. *et al.* The Pfam protein families database. *Nucleic Acids Res.* **32,** D138–D141 (2004).
32. Murzin, A. G., Brenner, S. E., Hubbard, T. & Chothia, C. SCOP: a structural classification of proteins database for the investigation of sequences and structures. *J. Mol. Biol.* **247,** 536–540 (1995).
33. Schellenberger, J., Park, J. O., Conrad, T. M. & Palsson, B. Ø. BiGG: a Biochemical Genetic and Genomic knowledgebase of large scale metabolic reconstructions. *BMC Bioinformatics* **11,** 213 (2010).
34. Bakan, A., Meireles, L. M. & Bahar, I. ProDy: protein dynamics inferred from theory and experiments. *Bioinformatics* **27,** 1575–1577 (2011).
35. McKinney, W. *Python for data analysis: Data wrangling with Pandas, NumPy, and IPython*. (‘ O’Reilly Media, Inc.’, 2012).
36. Cock, P. J. A. *et al.* Biopython: freely available Python tools for computational molecular biology and bioinformatics. *Bioinformatics* **25,** 1422–1423 (2009).
37. Humphrey, W., Dalke, A. & Schulten, K. VMD: visual molecular dynamics. *J. Mol. Graph.* **14,** 33–38 (1996).
38. Finn, R. D. *et al.* The Pfam protein families database. *Nucleic Acids Res.* gkp985 (2009).
39. Finn, R. D. *et al.* Pfam: the protein families database. *Nucleic Acids Res.* gkt1223 (2013).
40. Nanchen, A., Schicker, A. & Sauer, U. Nonlinear dependency of intracellular fluxes on growth rate in miniaturized continuous cultures of Escherichia coli. *Appl. Environ. Microbiol.* **72,** 1164–1172 (2006).
41. Megchelenbrink, W., Huynen, M. & Marchiori, E. optGpSampler: an improved tool for uniformly sampling the solution-space of genome-scale metabolic networks. *PLoS One* **9,** e86587 (2014).
42. Ebrahim, A., Lerman, J. A., Palsson, B. O. & Hyduke, D. R. COBRApy: COnstraints-Based Reconstruction and Analysis for Python. *BMC Syst. Biol.* **7,** 74 (2013).
